# Supplementary material for: Resolving the full spectrum of human genome variation using Linked-Reads
Source: Genome Res. 2019 Apr;29(4):635–45. doi: 10.1101/gr.234443.118 (PMC6442396; doi:10.1101/gr.234443.118)
Supplement: Supplemental Material [file supp_gr.234443.118_Supplemental_longranger-2.2.zip › longranger-2.2.tar.bz2!/lr2.2-public/lib/stan/cmdstan/stan/doc/api/html/struct_eigen_1_1internal_1_1general__matrix__vector__product_3_01_index_00_01stan_1_1agrad_1_1vacd54cabc78c49ca719650381b68b105e.html]

Stan: Eigen::internal::general\_matrix\_vector\_product< Index, stan::agrad::var, RowMajor, ConjugateLhs, stan::agrad::var, ConjugateRhs > Struct Template Reference


|  |  |
| --- | --- |
|  | Stan  2.5.0  probability, sampling & optimization |


- Main Page
- Related Pages
- Namespaces
- Classes
- Files

- Class List
- Class Index
- Class Hierarchy
- Class Members

All Classes Namespaces Files Functions Variables Typedefs Enumerations Enumerator Friends Macros Pages

- Eigen
- internal
- general\_matrix\_vector\_product< Index, stan::agrad::var, RowMajor, ConjugateLhs, stan::agrad::var, ConjugateRhs >

Public Types |
Static Public Member Functions |
List of all members

Eigen::internal::general\_matrix\_vector\_product< Index, stan::agrad::var, RowMajor, ConjugateLhs, stan::agrad::var, ConjugateRhs > Struct Template Reference

`#include <Eigen_NumTraits.hpp>`

|  |  |
| --- | --- |
| Public Types | |
| enum | { LhsStorageOrder = RowMajor } |
|  | |
| typedef stan::agrad::var | LhsScalar |
|  | |
| typedef stan::agrad::var | RhsScalar |
|  | |
| typedef scalar\_product\_traits  < LhsScalar, RhsScalar >  ::ReturnType | ResScalar |
|  | |

|  |  |
| --- | --- |
| Static Public Member Functions | |
| static EIGEN\_DONT\_INLINE void | run (Index rows, Index cols, const LhsScalar \*lhs, Index lhsStride, const RhsScalar \*rhs, Index rhsIncr, ResScalar \*res, Index resIncr, const RhsScalar &alpha) |
|  | |

## Detailed Description

### template<typename Index, bool ConjugateLhs, bool ConjugateRhs> struct Eigen::internal::general\_matrix\_vector\_product< Index, stan::agrad::var, RowMajor, ConjugateLhs, stan::agrad::var, ConjugateRhs >

Definition at line 197 of file Eigen\_NumTraits.hpp.

## Member Typedef Documentation

template<typename Index , bool ConjugateLhs, bool ConjugateRhs>

typedef stan::agrad::var Eigen::internal::general\_matrix\_vector\_product< Index, stan::agrad::var, RowMajor, ConjugateLhs, stan::agrad::var, ConjugateRhs >::LhsScalar

Definition at line 199 of file Eigen\_NumTraits.hpp.

template<typename Index , bool ConjugateLhs, bool ConjugateRhs>

typedef scalar\_product\_traits<LhsScalar, RhsScalar>::ReturnType Eigen::internal::general\_matrix\_vector\_product< Index, stan::agrad::var, RowMajor, ConjugateLhs, stan::agrad::var, ConjugateRhs >::ResScalar

Definition at line 201 of file Eigen\_NumTraits.hpp.

template<typename Index , bool ConjugateLhs, bool ConjugateRhs>

typedef stan::agrad::var Eigen::internal::general\_matrix\_vector\_product< Index, stan::agrad::var, RowMajor, ConjugateLhs, stan::agrad::var, ConjugateRhs >::RhsScalar

Definition at line 200 of file Eigen\_NumTraits.hpp.

## Member Enumeration Documentation

template<typename Index , bool ConjugateLhs, bool ConjugateRhs>

anonymous enum

| Enumerator | |
| --- | --- |
| *LhsStorageOrder* |  |

Definition at line 202 of file Eigen\_NumTraits.hpp.

## Member Function Documentation

template<typename Index , bool ConjugateLhs, bool ConjugateRhs>

|  |  |  |  |
| --- | --- | --- | --- |
| static EIGEN\_DONT\_INLINE void Eigen::internal::general\_matrix\_vector\_product< Index, stan::agrad::var, RowMajor, ConjugateLhs, stan::agrad::var, ConjugateRhs >::run | ( | Index | *rows*, |
|  |  | Index | *cols*, |
|  |  | const LhsScalar \* | *lhs*, |
|  |  | Index | *lhsStride*, |
|  |  | const RhsScalar \* | *rhs*, |
|  |  | Index | *rhsIncr*, |
|  |  | ResScalar \* | *res*, |
|  |  | Index | *resIncr*, |
|  |  | const RhsScalar & | *alpha* |
|  | ) |  |  |

inlinestatic

Definition at line 204 of file Eigen\_NumTraits.hpp.

---

The documentation for this struct was generated from the following file:

- src/stan/agrad/rev/matrix/Eigen\_NumTraits.hpp


---

[ Stan Home Page ]

*© 2011–2014,
Stan Development Team.*
